# An analysis of lecture video utilization in undergraduate medical education: associations with performance in the courses

**DOI:** 10.1186/1472-6920-9-6

**Published:** 2009-01-27

**Authors:** John A McNulty, Amy Hoyt, Gregory Gruener, Arcot Chandrasekhar, Baltazar Espiritu, Ron Price, Ross Naheedy

**Affiliations:** 1 Department of Cell Biology, Neurobiology and Anatomy, Stritch School of Medicine, Loyola University Chicago, Maywood, IL, USA; 2 Ralph P. Leischner Institute for Medical Education, Stritch School of Medicine, Loyola University Chicago, Maywood, IL, USA; 3 Office of Educational Affairs, Stritch School of Medicine, Loyola University Chicago, Maywood, IL, USA; 4 Department of Medicine, Stritch School of Medicine, Loyola University Chicago, Maywood, IL, USA; 5 Department of Neurology, Stritch School of Medicine, Loyola University Chicago, Maywood, IL, USA; 6 Information Technologies, Stritch School of Medicine, Loyola University Chicago, Maywood, IL, USA

## Abstract

**Background:**

Increasing numbers of medical schools are providing videos of lectures to their students. This study sought to analyze utilization of lecture videos by medical students in their basic science courses and to determine if student utilization was associated with performance on exams.

**Methods:**

Streaming videos of lectures (n = 149) to first year and second year medical students (n = 284) were made available through a password-protected server. Server logs were analyzed over a 10-week period for both classes. For each lecture, the logs recorded time and location from which students accessed the file. A survey was administered at the end of the courses to obtain additional information about student use of the videos.

**Results:**

There was a wide disparity in the level of use of lecture videos by medical students with the majority of students accessing the lecture videos sparingly (60% of the students viewed less than 10% of the available videos. The anonymous student survey revealed that students tended to view the videos by themselves from home during weekends and prior to exams. Students who accessed lecture videos more frequently had significantly (p < 0.002) lower exam scores.

**Conclusion:**

We conclude that videos of lectures are used by relatively few medical students and that individual use of videos is associated with the degree to which students are having difficulty with the subject matter.

## Background

Computer technologies have increasingly impacted medical education [1,2], most recently through the electronic distribution of videos, which are used extensively for a wide range of educational activities including demonstrating anatomical dissections [3], clinical procedures [4], assessments [4,5], providing access to online lectures [6] for use in asynchronous learning [6-10], as well as videoconferencing of patient scenarios [11]. In 2007, the Stritch School of Medicine instituted a policy that all lectures for the first two years of medical school would be provided online through secure networks using video streaming technology. The rationale for this policy was to provide additional asynchronous web-based resources for students to use for independent learning.

Several studies have investigated student satisfaction of lecture videos [12], attendance issues [7,13,14], educational efficiencies of videos [8,9,12,15], the effectiveness of videos as an instructional tool [8,9,15-17], as well as technical aspects [6,18]. The present study sought to provide additional information on student utilization of lecture videos in the undergraduate medical curriculum based on server log files supplemented by results from a student survey. To date, there is little detailed information on the use of lecture videos by medical students and the impact on their education.

The goal was to capture data on student use with minimal interventions under real-time curricular conditions. Specific questions included: How frequently do individual students view on-line videos of lectures? Are there differences between classes (freshman vs. sophomores) in the frequency of video viewing? To what degree do students view videos away from campus? To what degree are individual lectures viewed? Is there an association of the frequency of video viewing with performance on exams?

## Methods

The data were collected from first-year (M1; n = 143) and second-year (M2; n = 139) medical students at the Loyola University Chicago Stritch School of Medicine during the initial year that videos were made available for all lectures in the basic science courses.

### Collection and distribution of lecture videos

Collection of lecture videos in each room occurs through a dedicated workstation operating under Linux (Fedora version 8). The workstation contains an inexpensive video capture card (NTSC) which receives composite video and audio feeds from the lecture hall audiovisual equipment. A simple linux cron script uses video4linux to continuously capture video/audio in 30 minute segments daily from 7 a.m. to 9 p.m. Captured files are natively stored in an audio video interleave (AVI) format. A second cron script reprocesses all captured AVI files into corresponding Microsoft Windows Video (WMV) and MP3 audio files. Reprocessing of AVI files into WMV and MP3 files is accomplished using FFmpeg. FFmpeg is an open source software package that allows for the capture and conversion of video and audio files. In addition to conversion of the AVI files, the second cron script also transfers all WMV and MP3 files to the school's central video servers. Video servers (two) were Dell 2950s attached to 8TBs of storage operating under Microsoft Windows 2003 and Microsoft Media Services.

Access and distribution of the video occurs through the school's LUMEN  website. LUMEN's online course calendar was modified to provide direct hypertext links to captured videos and audios. Access to captured materials is restricted and users are required to provide their unique user id and password before files are made available. Detailed usages statistics are captured automatically.

### Data collection and analysis

Users must be authenticated to receive access to video and audio files which allows the system to capture detailed usage statistics. For each access the system collects user information (e.g., user name, workstation IP address, and browser type), session details (e.g., actual lecture date, time and faculty member) as well as actuall access date and time. This information is stored in a relational database that is utilized for usage analysis. Associations between the number of lectures viewed by individual students and their grade in the course were tested using the Student's t-test.

### Student survey

All students were offered an opportunity to respond to a survey (see Appendix), which was administered shortly after the study period was completed.

The study was exempted by the Institutional Review Board (IRB), Stritch School of Medicine.

## Results

**Figure 1:**
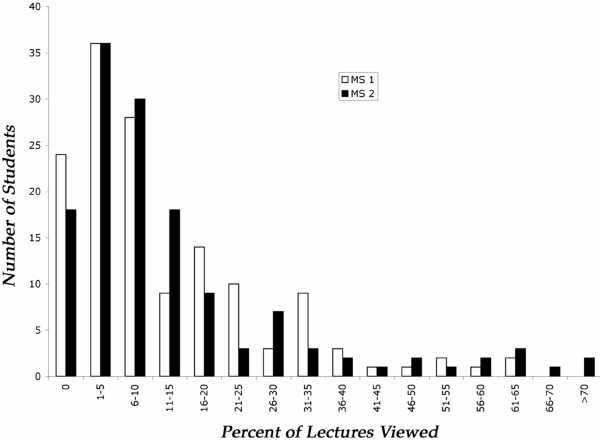
**Distribution of students in both classes according to the percent of lectures viewed**.

The majority of students viewed relatively few videos. Figure 1 shows that the distribution of the percent lectures viewed by students was highly skewed for both classes. The data revealed that 60% of M1 and M2 students viewed less than 10% of the available lectures. Only 5% of M1 and 9% of M2 students viewed more than 40% of the lectures and no student viewed more than 76% of the lectures in any course (Table 1). The data were converted to percent because the total number of lectures differed between classes (M1 = 49 lectures; M2 = 100 lectures).

**Table 1:** Metrics (means and ranges) for utilization of lecture videos from server log files.


	M 1	M 2

Percent of lectures viewed per student	12.6% (0-65%)	13.6% (0-76%)

Number of times individual lecture videos were viewed.	2.0 (1–4)	5.3 (2–14)

Number of students viewing individual lectures	17.3 (2–46)	17.7 (1–56)

Percent of videos viewed from school computers.	24%	33%

**Figure 2:**
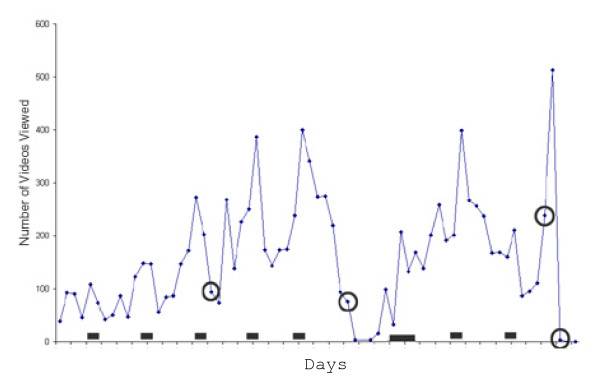
**Daily counts of videos viewed by MS2 students over the 10-week pathology/therapeutics course**. Exams dates are circled. The last exam was a separate final lab exam. Black bars indicate weekends (large black bar indicates Thanksgiving weekend).

The average number of lectures viewed per student, based on percent of total lectures, was similar for both classes (12.6% vs. 13.6%) (Table 1). M2 students tended to view any individual lecture about 2.5 times more often than M1 students (5.3 vs. 2.0 videos viewed per lecture per student) (Table 1). M2 students also tended to view more lectures from campus (33% of the time) compared to M1 students (24% of the time). Daily counts of the number of videos viewed showed that students tended to view videos over the weekend and prior to exams (Figure 2). The number of students viewing individual lectures was also relatively small (an average of 17–18 students viewed each lecture, Table 1), and 30% of all lectures were viewed by 10 students or less.

A total of 79 M1 students (53%) and 69 M2 students (47%) responded to the survey, which further elucidated how students used the videos (questions and student responses are provided in Appendix 1). Even with this 52% return (combined for both classes), there was generally good agreement between the student responses and the server log files regarding the frequency with which they viewed videos (i.e., 72% said they viewed 10 or fewer videos – Appendix 1) and the location where they viewed the videos (i.e., 33% stated they viewed videos from campus – Appendix 1). Students tended to view videos by themselves (82%) and used the videos primarily to fill in class notes and review for exams. The videos also were not used extensively by students to replace going to lectures (97% of respondents indicated that they used videos to replace going to less than 5 lectures). Reasons for using videos to replace going to lectures included the subject matter (17%), the lecturer (23%) and a larger number of "other" reasons (83%; e.g., car troubles, family issues, appointments, etc).

Another goal of our study was to determine if there were any associations between the level of video use and the performances of students related to grades in their courses. Since the total number of lectures differed between the two classes (M1 = 49 lectures; M2 = 100 lectures), we standardized the data to percent of lectures viewed (see Table 1). Because the means of final grades were similar for both classes (M1 = 85.1; M2 = 84.4; p = 0.42), the data were pooled and final grades divided into two groups according to the median percent of lectures viewed. Those students viewing the fewest lectures (0–7% of lectures; n = 136) had significantly (t = 3.17; p < 0.002) higher grades than those students viewing the most lectures (> 7% of lectures; n = 146). Similar trends were observed for each of the classes separately (M1; p = 0.03) (M2; p = 0.008). The availability of videos did not affect overall performance because average course grades did not differ significantly from the previous year when videos were unavailable for the same courses taken by M1's (t = 0.78; p = 0.43) and M2's (t = 0.53; p = 0.59).

## Discussion

An important finding from this study is that the majority of medical students utilized on-line lecture videos sparingly. Analysis of usage of lecture audio recordings in another study also showed that a relatively small subset of medical students used the resources extensively, while the majority (> 70%) accessed the audio files infrequently or not at all [19]. This wide disparity in student use of lecture files confirms earlier reports of high variability in student use of computer-aided instructional (CAI) applications [20-22]. In one study, as many as 45% of the students did not access supplemental CAI specifically developed for the curriculum [21].

A second important finding from our study was that student use of lecture videos appeared to be more closely associated with the degree to which students were having difficulty with the subject matter. It is noteworthy that in a another study, there was an inverse correlation between performance on in-course exams and length of time students logged into the server [23]. However, when specific supplemental CAI were evaluated, there were direct correlations between the degree to which computer resources were utilized by individual students and their performances on exams [21,24]. An important difference is that CAI typically supplements the curriculum whereas use of on-line videos evaluated in the present study duplicate the curriculum.

Aggregate analyses obviously do not address benefits that individual students may have received from the video resources, which is a limitation of the study. In one study designed to compare test performance of video vs. live lectures the authors found that students who were provided video lectures had better or similar exam scores compared to students who attended live lectures [16]. Although our study did not directly address the effects of lecture videos on performances in the courses, there appears to have been no effect because average grades in the courses were not statistically different from the prior year when lecture videos were not available.

By combining server log files with student surveys we were able to glean information on the nature of student usage of video files. Our finding that students tended not to use videos to replace going to lectures was consistent with a recent study [7] revealing that medical students made deliberate decisions about lecture attendance (e.g., experiences with particular teachers, subject of lecture), and their attendance was not influenced by the availability of electronic resources. The present study has extended our understanding of how medical students use lecture videos by showing that students tend to view the videos alone for clarifying their notes on weekends and/or reviewing for exams. However, another limitation of the study is the large number of students who elected not to participate in the survey. Accordingly, we were unable to accurately determine the degree to which student attendance at lectures had changed.

Literature addressing the pedagogy of implementing and evaluating computer technologies in medical education increasingly emphasizes the need to better understand how learners are interacting with the applications [1,2,25]. Anticipating that videos will continue to play a role in paradigm shifts of medical curricula, several questions still remain. Does the level of individual student use of videos vary from course to course? Will utilization increase as videos become further integrated within the curriculum? How frequently do students review lecture videos from earlier classes in preparing for board exams? In addition, can video logs be used for curricular revisions by identifying specific content areas that need to be expanded or eliminated? To what extent are faculty reviewing lectures, and can videos be used effectively for faculty development?

## Conclusion

The results of our study demonstrated that significant use of lecture videos in the medical curriculum was limited to a relatively small percentage of students, who tended to view videos by themselves to fill in notes and review. Attendance at lectures did not seem to be significantly affected. The frequency with which individual students viewed videos was inversely associated with their grades in the courses.

## Appendix

**Appendix 1**. Responses of students to survey about lecture videos.

How frequently did you view videos for lectures?

   Never               12%

   1–5 lectures            31%

   6–10 lectures            33%

   11–20 lectures            12%

   More than 20 lectures         12%

If you viewed videos of lectures, (check all that apply)

   It replaced going to lecture      42%

   I used it to complete my notes   86%

   I used it to review for exams.   64%

If you used videos to review for exams, how useful were they?

   Not useful            0%

   Somewhat useful         52%

   Very useful            48%

If you used the videos to replace going to lecture, how many lectures did they replace?

   Not applicable         53%

   1–5 lectures            44%

   6–10 lectures            2%

   11–20 lectures            1%

   More than 20 lectures         1%

If you used the videos to replace going to lecture, it was because: (check all that apply):

   The subject matter could be learned by reading and/or handouts   17%

   The lecturer was bad         23%

   Other reason            83%

Where did you mostly view the videos?

   Home               67%

   School               33%

   Other               0%

How many videos did you view with other students?

   None               82%

   1–5 videos            15%

   6–10 videos            2%

   11–20 videos            1%

   > 20 videos            0%

Do you think you will use the lecture videos to study/review for boards?

   Yes               44%

   No               56%

Was the quality of video adequate?

   Terrible            9%

   OK, but could be improved      62%

   Fine               26%

Great               3%

## Competing interests

The authors declare that they have no competing interests.

## Authors' contributions

JAM originated the idea for the research, oversaw the collection and analysis of data, and wrote the manuscript. AH assisted with the collection and analysis of data and helped revise the manuscript. GG helped with interpretation of data and writing the manuscript. AC assisted with collection of data and revising the manuscript. BE contributed to the manuscript. RP coordinated collection of the data and helped revise the paper. RN developed applications for collecting the data. All authors have read and approved the final manuscript.

## Pre-publication history

The pre-publication history for this paper can be accessed here:


